# Description of two deep-water copepods of the genus *Leptotachidia* Becker from the northwestern Pacific (Harpacticoida, Pseudotachidiidae)

**DOI:** 10.3897/zookeys.873.34630

**Published:** 2019-08-29

**Authors:** Jong Guk Kim, Ok Hwan Yu, Jimin Lee

**Affiliations:** 1 Marine Ecosystem Research Center, Korea Institute of Ocean Science & Technology, Busan 49111, Korea Korea Institute of Ocean Science & Technology Busan South Korea

**Keywords:** abyssal harpacticoids, biodiversity, Brodskaya organ, Danielsseniinae, deep-sea, *Paradanielssenia* group

## Abstract

The monospecific genus *Leptotachidia* Becker, 1974 (Pseudotachidiidae Lang, 1936) was previously known only from the deep Atlantic. Female specimens of two unknown species of this genus were collected from abyssal sediments during an expedition to the northwestern Pacific on board research vessel *ISABU* (Korea Institute of Ocean Science and Technology) in November 2017. In this paper we describe the females of two new species, *L. senaria***sp. nov.** and *L. apousia***sp. nov.** The new species were attributed to the genus *Leptotachidia* by the combination of the five-segmented female antennule, presence of the Brodskaya organ on the distal antennulary segment, and the second exopodal segments of second to fourth legs without inner setae. An outstanding character of both Pacific species is the reduced armature of thoracic legs in contrast to the type species, *L. iberica* Becker, 1974. In addition, the setal armature of *L. senaria***sp. nov.** and *L. apousia***sp. nov.** is unique within the genus in that the female leg 5 of *L. senaria***sp. nov.** has six elements instead of five; and the antennary exopod of *L. apousia***sp. nov.** bears a single seta on the proximal segment instead of two. This is the first record of *Leptotachidia* from the Pacific. A key to all three species of *Leptotachidia* is provided.

## Introduction

Despite their abundance and high biomass in deep-sea meiobenthic assemblages, little is known on the diversity of abyssal harpacticoids and more than 95% of them remain undescribed (Seifried 2004). Most taxonomic studies on abyssal harpacticoids have been performed in the Atlantic (Seifried 2004) and a limited number of papers are available on the deep-sea harpacticoid fauna from the Pacific (e.g. Itô 1982, 1983; Humes and Voight 1997; Huys and Conroy-Dalton 1997; Lee and Yoo 1998; Conroy-Dalton and Huys 1999, 2000; Lee and Huys 1999, 2000; López-González et al. 2000; Gollner et al. 2008; Back et al. 2010; Cho et al. 2016; Gómez and Díaz 2017; Vakati et al. 2017; Gómez 2018a-c; George and Schwabe 2019).

In the northwestern Pacific, 29 harpacticoid copepods belonging to the families Aegisthidae Giesbrecht, 1893, Ectinosomatidae Sars, 1903, Tegastidae Sars, 1904, Pseudotachidiidae Lang, 1936, Normanellidae Lang, 1944, Argestidae Por, 1986, and Parameiropsidae Corgosinho & Martínez Arbizu, 2010 have been reported from bathyal benthic and hyperbenthic habitats, such as trenches, cold seeps, and hydrothermal vents (Itô 1982, 1983; Lee and Yoo 1998; Lee and Huys 1999, 2000; Back et al. 2010; George and Schwabe 2019). Although the species of Pseudotachidiidae are important members in the deep-sea assemblage (Willen and Schulz 2007; Willen 2008, 2009; Kitahashi et al. 2012; George et al. 2014), three members of the genus *Pseudotachidius* Scott T., 1898 have been recorded only from the deep sea off Mindanao in the Philippines (Itô 1983).

Specimens of the family Pseudotachidiidae were collected from the deep sea in the northwestern Pacific during an oceanographic cruise on board RV *ISABU* of the Korea Institute of Ocean Science and Technology (KIOST). This paper deals with the second and third member of the genus *Leptotachidia* Becker, 1974. We provide the full description and detailed illustrations of the two new species. Additionally, a key to species of the genus is given. This is the first record of *Leptotachidia* from the Pacific.

## Materials and methods

**Figure 1.**
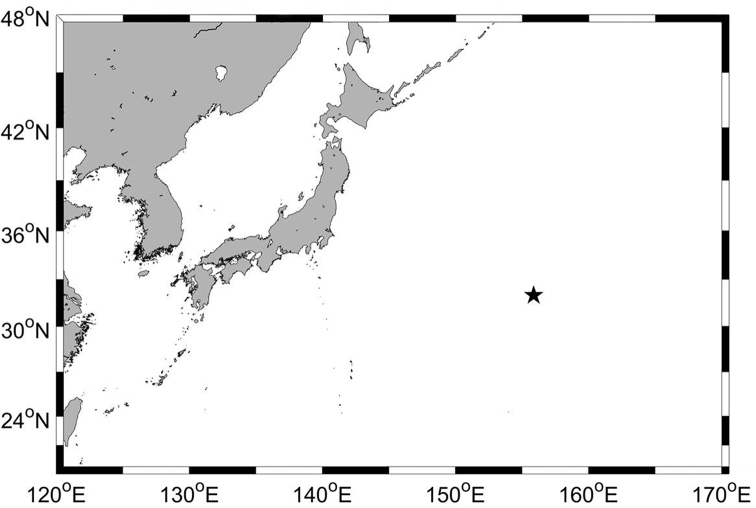
Map showing the sampling station.

**Figure 2.**
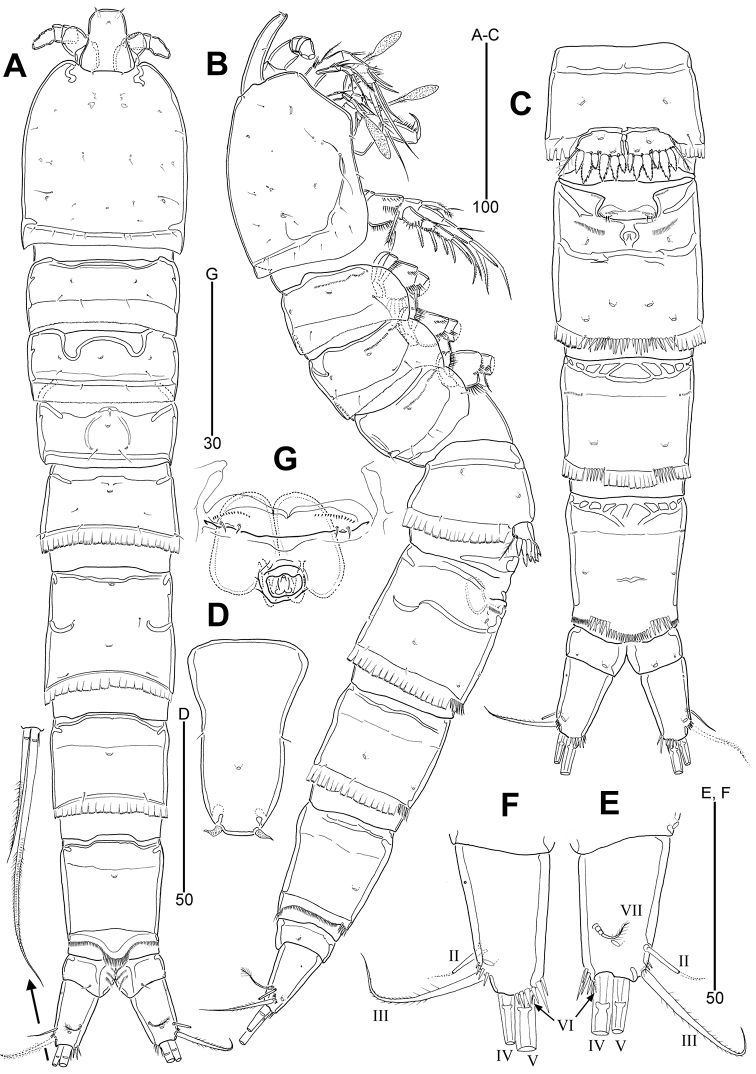
*Leptotachidia senaria* sp. nov., female **A** habitus, dorsal **B** habitus, lateral **C** urosome, ventral **D** rostrum, dorsal **E** caudal ramus, dorsal **F** caudal ramus, ventral **G** genital field, ventral.

**Figure 3.**
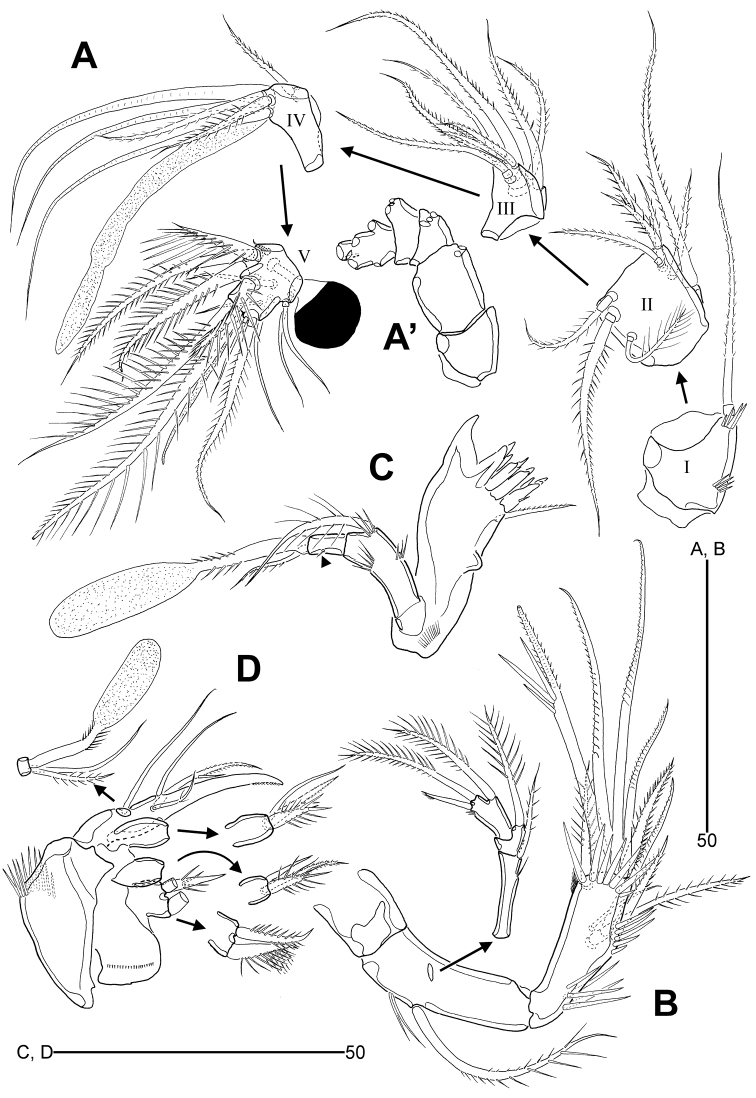
*Leptotachidia senaria* sp. nov., female **A** antennule **B** antenna **C** mandible (arrowhead indicates a rudimental lateral seta) **D** maxilla.

**Figure 4.**
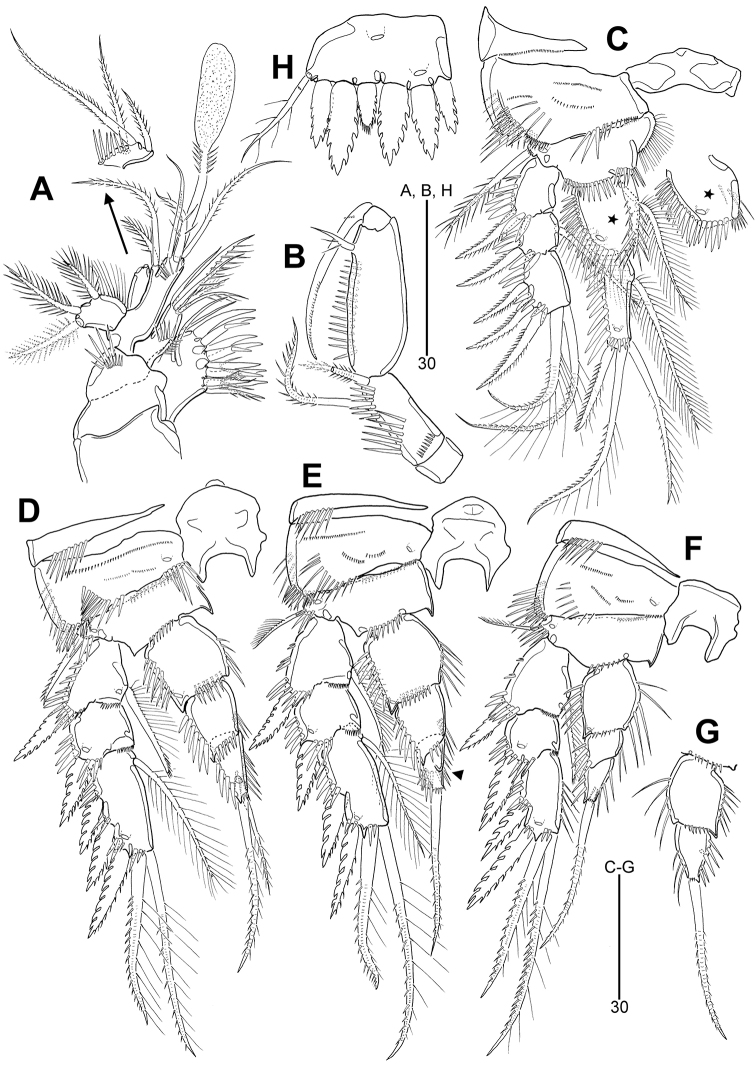
*Leptotachidia senaria* sp. nov., female **A** maxillule **B** maxilliped **C** P1, anterior **D** P2, anterior **E** P3, anterior (arrowhead showing the modified and dented inner margin) **F** P4, anterior **G** abnormality of P4 endopod, anterior **H** P5, anterior.

**Figure 5.**
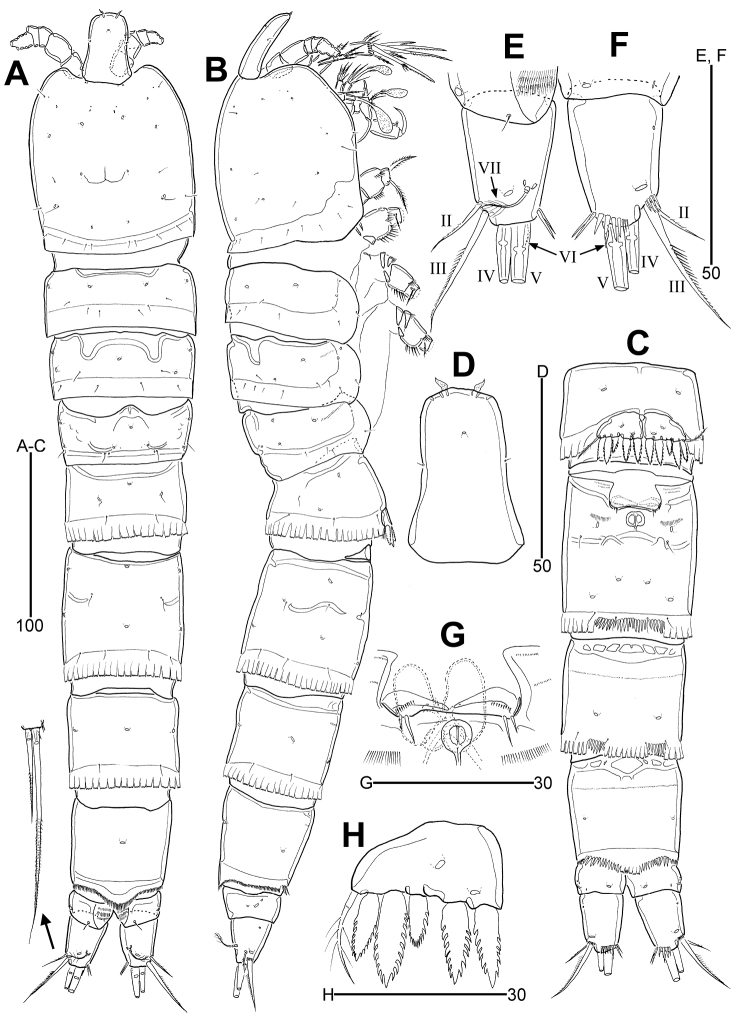
*Leptotachidia apousia* sp. nov., female **A** habitus, dorsal **B** habitus, lateral **C** urosome, ventral **D** rostrum, dorsal **E** caudal ramus, dorsal **F** caudal ramus, ventral **G** genital field, ventral **H** P5, anterior.

**Figure 6.**
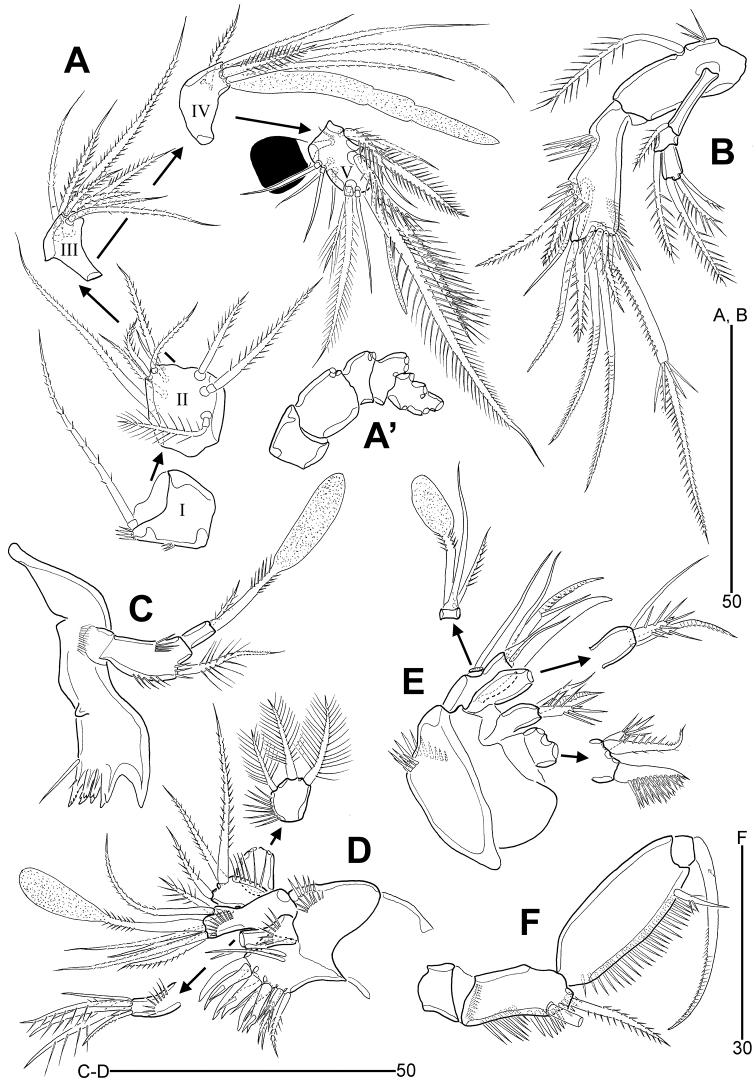
*Leptotachidia apousia* sp. nov., female **A** antennule **B** antenna **C** mandible **D** maxillule **E** maxilla **F** maxilliped.

**Figure 7.**
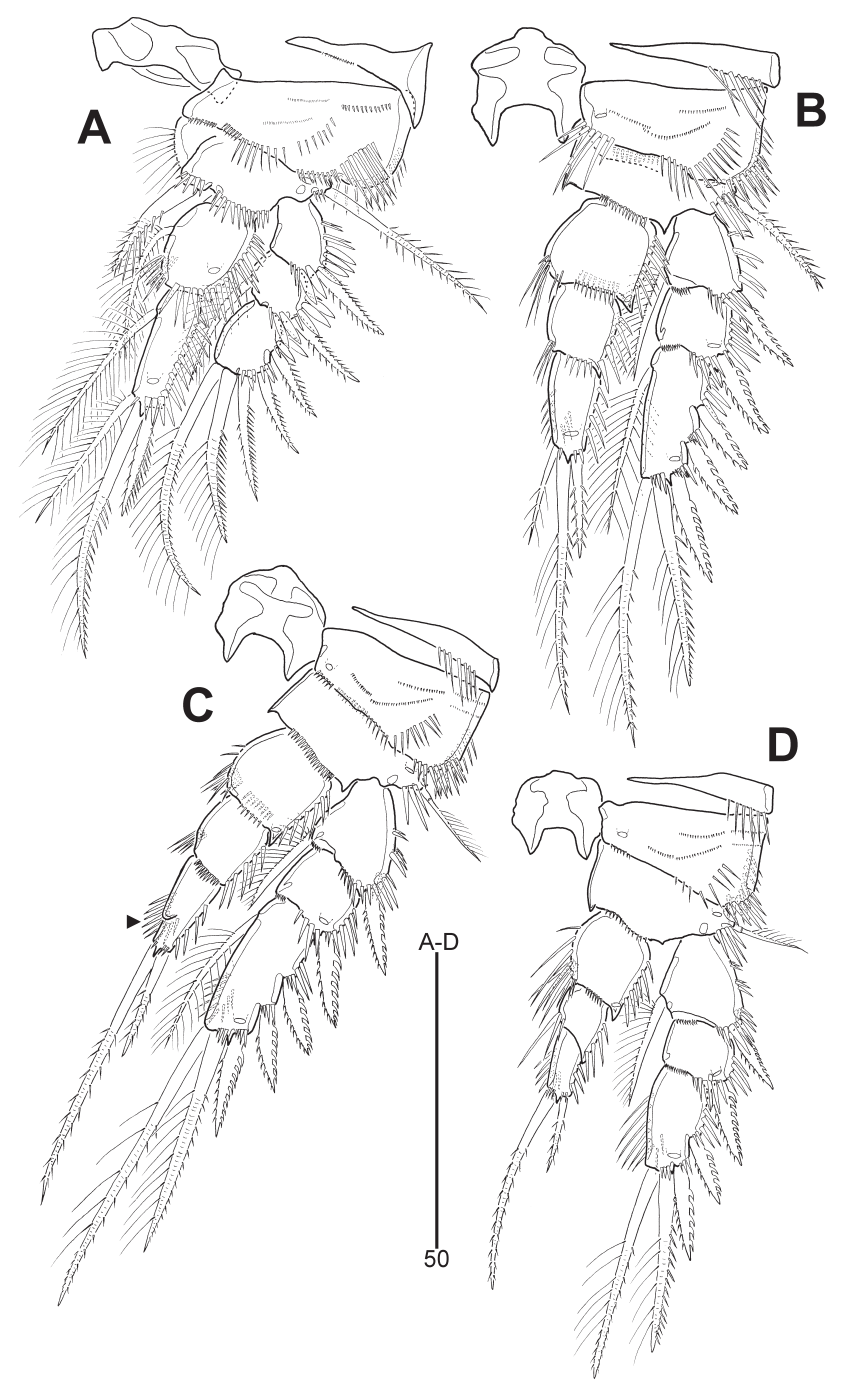
*Leptotachidia apousia* sp. nov., female **A** P1, anterior **B** P2, anterior **C** P3, anterior (arrowhead showing the modified and dented inner margin) **D** P4, anterior.

A KIOST cruise of the research vessel RV *ISABU* in November 2017 explored the deep-sea benthic fauna of the northwestern Pacific. As a result of this survey, two new species, *Leptotachidia senaria* sp. nov. and *L. apousia* sp. nov., were found from one sampling location (Fig. 1). Samples of abyssal sediments were taken using a multiple corer equipped with eight acrylic cores of 10 cm inner diameter. The upper layer (>5 cm) of each core was separately preserved in a 10 % formalin solution for quantitative or qualitative analyses. In the laboratory, meiofauna was removed from the sediments using the Ludox HS-40 method (Burgess 2001). Harpacticoids were sorted under a dissecting microscope (Leica M165 C, Germany). For taxonomic examination, the reverse slide method of Humes and Gooding (1964) was adopted. Animals were prepared in lactic acid on the reverse slide. All drawings of type specimens were made with the aid of a drawing tube under a differential interference contrast (DIC) microscope (Leica DM2500, Germany). The habitus of the material presented here was first depicted, and their appendages and urosome were drawn after dissection with tungsten needles. After examination, appendage and urosome were mounted in lactophenol on an H-S slide each (Shirayama et al. 1993). Type material was deposited in the Marine Biodiversity Institute of Korea (MABIK). We followed the descriptive terminology of Huys and Boxshall (1991). Scale bars in Figures 2–7 are given in μm.

Abbreviations used in text and figures are as follows:

**ae** aesthetasc;

**P1–P6** first to sixth thoracic legs;

**Exp (enp)-1 (-2,-3)** proximal (middle, distal) segment of exopod (endopod).

## Systematics

### Family Pseudotachidiidae Lang, 1936

#### Subfamily Danielsseniinae Huys & Gee in Huys et al., 1996

##### Genus Leptotachidia

Taxon classificationAnimaliaHarpacticoidaPseudotachidiidae

Becker, 1974

D92339C9EDF95EEB8FE9B3826D259881

###### Type species.

*Leptotachidia iberica* Becker, 1974

##### Leptotachidia senaria sp. nov.

Taxon classificationAnimaliaHarpacticoidaPseudotachidiidae

E666695AE8BD5C4382218483586A8EC9

http://zoobank.org/076F631E-0D2C-4F7B-B8C7-29F56BFD8C79

Figs 2 , 3 , 4

###### Type locality.

Abyssal basin of the Northwest Pacific Ocean (31°58'42.9"N, 155°53'42.7"E), 5482 m depth (Fig. 1).

###### Material examined.

Holotype: adult female dissected and mounted on 11 slides (cat. no. MABIK CR00246484) collected from the type locality on 1 November 2017.

###### Etymology.

The specific epithet is derived from the Latin *senaria* meaning ‘consisting of six’ and refers to the female fifth leg with six elements in both rami, which is a unique characteristic within the genus *Leptotachidia*. It is in the nominative singular. Gender feminine.

###### Description of female.

Total body length about 710 μm measured from anterior tip of rostrum to posterior margin of caudal rami in lateral view; greatest width about 106 μm measured at the middle of cephalothorax. Habitus (Fig. 2A, B) elongate, cylindrical, with weak constriction between prosome and urosome; urosome slightly narrower than prosome. Prosome (Fig. 2A, B) composed of cephalothorax and three free pedigerous somites. Cephalothorax bell-shaped, slightly longer than wide in dorsal view, as long as 21% of total body length; surface ornamented with several small and large pores, and short and long sensilla; hyaline frill smooth; arthrodial membrane of first pedigerous somite visible posteriorly. Metasome gradually narrowing posteriorly; second pedigerous somite with two, third and fourth pedigerous somites with a single mid-dorsal pore, all free pedigerous somites with paired large pores and sensilla dorsally and laterally, with two lateral rows of minute spinules; pleural areas of second and third pedigerous somites distinctly produced posteriorly, weak in fourth one; hyaline frills of second and third pedigerous somites well developed and weakly serrate, smooth in fourth one. Dorsal surface ridges on third and fourth pedigerous somites partly modified with internal chitinous ribs (Fig. 2A).

Urosome (Fig. 2A–C) gradually narrowing posteriorly, with large pores and paired sensilla dorsally and laterally; first urosomite with deeply incised and continuous hyaline frill, of genital double-somite interrupted ventrally by continuous row of strong spinules, of second abdominal somite interrupted ventrally by two short rows of strong spinules. Arthrodial membranes of all urosomites visible posteriorly. Original division between genital somite and third urosomite indicated by ventral and lateral subcuticular ridges, but dorsal aspect fused. Genital field with a transverse genital slit (Fig. 2G) covered by single plate with P6 represented by two vestigial setae and one row of minute spinules on both sides; copulatory pore located in a depression posterior to genital slit; seminal receptacles paired, placed inside genital aperture and connected with copulatory pore; one row of spinules and one large pore on ventrolateral surface; two pairs of large ventral pores on first abdominal somite. Second abdominal and penultimate somites with particular polygonal ribs along anteroventral surface (Fig. 2C). Penultimate somite with pseudoperculum, the latter with long spinules; posterior margin finely dentate dorsally and laterally, coarsely dentate ventrally, with two rows of stout spinules ventrolaterally. Anal somite short, with one pair of sensilla dorsally, one lateral pore, and one small and one large pores ventrally; dorsal surface depressed medially forming anal opening, with three rows of setules on inner margin.

Caudal rami (Fig. 2A–C, E, F) divergent, slightly tapering towards posterior end, about 1.7 times as long as greatest basal width, with one dorsal and one ventrolateral large pore; rows of spinules present near base of setae III and V, and on inner distal corner; a small pore located at proximal forth of ventrolateral surface; with six caudal setae: seta I absent; seta II bare, small, at distal forth dorsolaterally; seta III pinnate, about three times as long as seta II, and inserted in lateral margin subdistally; principal setae IV and V well developed, distally pinnate and rat tail-like, seta V about twice as long as seta IV; seta VI minute, inserted at inner distal corner; seta VII tri-articulate at base, arising from distal third of inner-dorsal surface, with fine hairs distally.

Rostrum (Fig. 2D) large, bell-shaped, with one median pore, one pair of medial sensilla, and one pair of subdistal sensilla modified into aesthetascs-like structures.

Antennule (Fig. 3A) short, five-segmented; segment I with one long distal pinnate seta and two rows of stout outer spinules; segment II largest, about 1.2 times as long as wide, with one plumose and eight pinnate setae; segment III with eight pinnate setae; segment IV smallest, with three long bare and three pinnate setae, and one aesthetasc fused basally to adjacent one long bare seta; segment V with one pinnate, six spinulose and seven bare setae, one aesthetasc fused basally to adjacent two spinulose setae, and one densely opaque bulbous appendage (Brodskaya organ). Segment V with apical acrothek. Armature formula as follows: 1-[1] , 2-[9], 3-[8], 4-[5 + (ae + 1)], 5-[12 + Brodskaya organ + acrothek].

Antenna (Fig. 3B). Coxa distinct, unornamented. Allobasis with one long pinnate seta and one group of long spinules on abexopodal margin. Exopod three-segmented; proximal segment elongate, 4.5 times as long as wide, with one bipinnate and one minute bare setae; middle segment small, with one spinulose seta; distal segment with two spinulose setae and one seta with distal part uniserrate. Free endopod with two rows of stout spinules laterally; lateral armature comprising one bare, one geniculate and two bipinnate setae; distal armature composed of two bipinnate and four geniculate setae, one of which bears spinules medially; distal margin with one row of stout spinules and one subdistal surface frill.

Mandible (Fig. 3C). Coxa with one medial protuberance ventrally and one row of setules near base of palp; gnathobase with one large uni-cuspidate and four multi-cuspidate teeth, and one unipinnate ventral seta. Palp uniramus, two-segmented; basis elongate, with three rows of spinules and one plumose seta; endopod small, one-segmented, with one small unipinnate seta and a claviform aesthetasc with peduncle partially bipinnate distally, and one rudimentary seta (indicated by arrowhead in Fig. 3C) on lateral margin.

Maxillule (Fig. 4A). Praecoxa with two rows of outer spinules; arthrite with nine distal elements and two surface setae anteriorly. Coxal endite with one anterior row of spinules, one lateral row of setules, and one bare, one plumose and three unispinulose distal setae. Basis with two endites; distal endite largest, with one bare, one plumose and one pinnate setae, and a claviform aesthetasc with peduncle partially bipinnate distally, and one row of anterior spinules; subdistal endite with one long and one small bipinnate setae. Exopod broad, one-segmented, with three spinulose apical setae and one row of inner setules. Endopod one-segmented, with three bipinnate setae and one row of outer spinules.

Maxilla (Fig. 3D). Syncoxa with one transverse row of stout outer spinules and one row of minute surface spinules; with three endites: proximal endite bilobate, with one stout spinulose seta on proximal lobe, and one bipinnate and one spinulose setae on distal lobe; medial and distal endites with one bare, one bipinnate and one spinulose setae each. Allobasis with one long bare seta near base of endopod, drawn into a weakly pinnate claw bearing one bare seta, one pinnate element with an accessory spinule, and one weakly plumose seta. Endopod small, one-segmented, with one bare and one bipinnate setae, and a claviform aesthetasc with peduncle partially bipinnate.

Maxilliped (Fig. 4B). Praecoxa small, unornamented. Coxa elongate, with two pinnate geniculate setae subdistally, with two inner rows of stout spinules and one row of minute spinules proximally. Basis elongate, ovoid, with one row of stout inner spinules and one subdistal setulose seta. Endopod one-segmented, represented by a dentate claw bearing one vestigial accessory seta.

P1 (Fig. 4C). Praecoxa small, with one row of minute spinules distally. Intercoxal sclerite wide, slightly arched and unornamented. Coxa large, medially with three rows of minute spinules and one row of long spinules anteriorly; distally with one row of minute spinules and one row of stout spinules anteriorly; with two rows of spinules near outer distal corner; with one row of posterior spinules. Basis with one row of inner setules; with one row of stout spinules on distal pedestal of endopod; with one large pore near outer seta anteriorly; outer seta long, spinulose and arising from peduncle with three minute spinules; inner spine stout, spinulose and arising from peduncle bearing one row of spinules. Exopod three-segmented, reaching middle of enp-2; each segment with strong outer and distal spinules; exp-1 with one bipinnate outer spine; exp-2 with one bipinnate outer spine and one plumose inner seta; exp-3 with three bipinnate outer spines, and two rat tail-like setae with outer spinules and inner setules. Endopod longer than exopod, two-segmented; each segment with outer and distal spinules; enp-1 broad, with one plumose inner seta, with one large anterior pore, one row of posterior spinules, and one row of inner distal setules; enp-2 elongate, about 2.7 times as long as wide, with one pinnate distal outer spine, and two setae with pinnate outer and setulose inner margins.

P2–P4 (Fig. 4D–F). Praecoxa small, as wide as coxa, with one row of stout distal spinules. Intercoxal sclerite developed, concave distally, with two distal pointed projections. Coxa broad, with three rows of minute medial spinules, one row of longer spinules and one large pore anteriorly; one row of stout outer distal spinules; with one row of outer spinules and one row of distal spinules posteriorly; with one row of stout (P2) or minute (P3–P4) inner distal spinules anteriorly. Basis with one large anterior pore, one row of distal spinules on pedestal of endopod, and one row of spinules near base of outer seta; the latter unipinnate (P2) or uniplumose (P3–P4); outer setae of P3–P4 with internal fracture plane; inner distal corner produced. Exopod slightly longer than endopod, three-segmented; each segment with outer and distal spinules; exp-1 and exp-2 with inner distal frills; P4 exp-3 with one row of inner setules; anterior pores on all exopodal segments of P2, and exp-2 and exp-3 of P3–P4; exp-1 with one serrate outer spine and one uniplumose inner seta; exp-2 with one serrate outer spine; exp-3 with two (P3–P4) or three (P2) serrate outer spines, two rat tail-like distal setae with outer marine spinulose, and inner margin setulose, and with one plumose inner proximal seta (P2–P3), or without inner armature (P4). Endopod tapering distally, three-segmented; each segmented with outer, inner and distal spinules; with distal posterior spinules in P2–P3 enp-1; with anterior pores on P2–P3 enp-1 and P2 enp-3; all endopodal segments without inner element; P2 enp-3 with one pinnate, rat tail-like and one plumose setae; P3–P4 enp-3 with one pinnate, rat tail-like seta; inner margin of P3 enp-3 modified and dented (indicated by arrowhead in Fig. 4E). P1–P4 armature formulae:

**Table:** 

	Exopod	Endopod
P1	0.1.023	1.121
P2	1.0.123	0.0.020
P3	1.0.122	0.0.010
P4	1.0.022	0.0.010

P5 (Figs 2C, 4H). Small sclerite present between both members. Exopod and baseoendopod fused, forming a single plate, with five coarsely serrate and one finely serrate distal spines; with two large anterior pores; outer seta plumose, arising from small peduncle and with internal fracture plane.

###### Male.

Unknown.

###### Variability.

Left P4 of the female holotype normal, right endopod of P4 with two segments (Fig. 4G).

##### Leptotachidia apousia sp. nov.

Taxon classificationAnimaliaHarpacticoidaPseudotachidiidae

95C542841D0352D38A64FD69385637FB

http://zoobank.org/D254F962-9EB5-4A3B-98F5-6E04BA0F295A

Figs 5 , 6 , 7

###### Type locality.

Abyssal basin of the Northwest Pacific Ocean (31°58'42.9"N, 155°53'42.7"E), 5482 m depth (Fig. 1).

###### Material examined.

Holotype (adult female) dissected and mounted on 11 slides (cat. no. MABIK CR00246485) collected from the type locality on 1 November 2017.

###### Etymology.

The species name is derived from the Greek απουσία, *apousia*, meaning absence, lacking, and alludes to the absence of an inner seta in P3 enp-3. It is in the nominative singular. Gender feminine.

###### Description of female.

Total body length about 608 μm, greatest width about 99 μm measured at the middle of cephalothorax. Habitus (Fig. 5A, B) slightly damaged (thoracic legs detached), as in *L. senaria* sp. nov., but slightly narrower; ornamentation of somites slightly different from *L. senaria* sp. nov. in number and position of both sensilla and pores.

Cephalothorax (Fig. 5A, B) about 1.2 times as long as wide in dorsal view, as long as 18 % of total body length. Pleural areas of free pedigerous somites more weakly developed than in *L. senaria* sp. nov. Hyaline frills of second to fourth pedigerous somites smooth.

Urosome (Fig. 5A–C) about 1.1 times as long as prosome. With deeply incised hyaline frills as in *L. senaria* sp. nov. except for penultimate somite with weakly incised hyaline frill ventromedially. Genital double-somite divided by lateral and ventral ridges, but fused dorsally and ventrolaterally. Genital slit (Fig. 5G) covered by single plate with two minute setae, inner about twice as long as outer, and one row of minute spinules on both sides; copulatory pore posterior to genital slit; seminal receptacles smaller than in *L. senaria* sp. nov. Pseudoperculum (Fig. 5A) on penultimate somite slightly wider than in *L. senaria* sp. nov. Anal somite small, with one pair of sensilla, one large and two small lateral pores, and one large and one small ventral pores; anal opening fringed with three pairs of setular rows.

Caudal rami (Fig. 5A–C, E, F) slightly shorter than in *L. senaria* sp. nov., about 1.4 times as long as greatest width, with one large pore anteriorly and posteriorly; ventral spinule rows present near base of seta III and seta V, and closed to inner distal corner; armature as in *L. senaria* sp. nov. except for seta V about 2.8 times as long as seta IV.

Rostrum (Fig. 5D) as in *L. senaria* sp. nov. except for a pair of aesthetasc-like sensilla on distal margin closer to each other than in *L. senaria* sp. nov.

Antennule (Fig. 6A) as in *L. senaria* sp. nov. except for arrangement of elements on segments II, IV and V; segment II 1.1 times as long as wide, with one plumose, one bare and seven pinnate setae; segment IV with two pinnate and four weakly pinnate setae, and one aesthetasc fused to adjacent long weakly pinnate seta; segment V with one weakly bipinnate, two plumose, five spinulose and six bare setae; with distal acrothek composed of one aesthetasc and adjacent two spinulose setae, and one densely opaque bulbous appendage (Brodskaya organ). Armature formula as follows: 1-[1], 2-[9], 3-[8], 4-[5 + (ae + 1)], 5-[12 + Brodskaya organ + acrothek].

Antenna (Fig. 6B) as in *L. senaria* sp. nov. except for exp-1 with only one plumose seta.

Mandible (Fig. 6C) as in *L. senaria* sp. nov. except for absence of a rudimentary seta on lateral margin.

Maxillule (Fig. 6D), maxilla (Fig. 6E), and maxilliped (Fig. 6F) as in *L. senaria* sp. nov.

General shape of P1–P4 (Fig. 7A–D) as in *L. senaria* sp. nov. except for setal armatures of P3 exp-3 and P2–P4 enp-3; P3 exp-3 (Fig. 7C) with three outer spines; P2 enp-3 (Fig. 7B) with one small bipinnate outer spine, one long apical seta with outer spinules and inner setules, and one small plumose apical seta; P3–P4 enp-3 (Fig. 7C–D) with one small pinnate outer spine and one long bipinnate apical seta; inner margin of P3 enp-3 modified and dented as in *L. senaria* sp. nov. (indicated by arrowhead in Fig. 7C).

P1–P4 armature formulae:

**Table:** 

	Exopod	Endopod
P1	0.1.023	1.121
P2	1.0.123	0.0.111
P3	1.0.123	0.0.011
P4	1.0.022	0.0.011

General structure of P5 (Fig. 5H) as in *L. senaria* sp. nov. except for setal armature; distal margin with one finely serrate and four coarsely serrate spines.

###### Male.

Unknown.

## Discussion

The series of papers by Gee and Huys (1990, 1991, 1994) and Huys and Gee (1992, 1993, 1996a, b) contributed to defining the phylogenetic relationships among the danielsseniin genera and to establishing several new genera of the family Paranannopidae Por, 1986 (= Danielsseniinae Huys and Gee in Huys et al. 1996; *cf.*Huys 2009). The presence of aesthetascs on the mouthparts, which character is unique within other harpacticoid families and its position is homologous, is a significant synapomorphy for a core of genera, *Jonesiella* Brady, 1880, *Paradanielssenia* Soyer, 1970, *Leptotachidia* Becker, 1974, *Micropsammis* Gee & Huys, 1991, *Telopsammis* Gee & Huys, 1991, *Peltisenia* Huys & Gee, 1996, *Sentiropsis* Huys & Gee, 1996, and *Nyxis* Willen, 2009 (Gee and Huys 1991; Willen 2005, 2008, 2009; Willen and Schulz 2007; Kim et al. 2011).

The danielsseniin *Paradanielssenia* group composed of *Paradanielssenia*, *Leptotachidia*, *Micropsammis* and *Telopsammis* shares the aesthetascs on the mouthparts modified into claviform stuructures (Gee and Huys 1991; Willen 2008, 2009). Willen (2008, 2009) suggested that the genera of the *Paradanielssenia* group are more derived than the other congeners given four autapomorphies: (1) the loss of the mandibular exopod; (2) reduced setation of the mandibular basis (less than three setae); (3) the presence of a rigid apophysis on the male P2 enp-3 instead of an outer seta, except for *Leptotachidia*; and (4) the comparatively shorter innermost seta on the female P5 baseoendopod.

Three genera, *Leptotachidia*, *Micropsammis* and *Telopsammis*, seem to be more derived than *Paradanielssenia* and share the following synapomorphies: (1) presence of deeply incised hyaline frills on urosomites; (2) presence of an elongate mandibular basis with one seta; (3) presence of rat tail-like terminal setae on both rami of P1; (4) armature complement of P2–P4 reduced, for example the presence of only two outer spines on P4 exp-3, the absence of inner setae on P2 endopod, P3 enp-1 and enp-3, and P4 enp-3; and (5) fusion of both rami of P5 in both sexes (Gee and Huys 1991; Mielke 1997; Willen 2008). The presence of three basal setae in mandible is a primitive character and indicates that *Paradanielssenia* is situated in a basal position within this lineage.

The genus *Leptotachidia* is closely related to *Telopsammis* (Gee and Huys 1991; Huys 2009), although there is a marked difference in the sexual dimorphism of the male P2 endopod between these two genera (the outer element of distal segment is developed into an apophysis in the male of the latter genus except the absence of this modification in *T. pelobionta* Kornev & Chertoprud, 2008) (Mielke 1975, 1997; Gee and Huys 1991; Kornev and Chertoprud 2008). The sister group relationship between *Leptotachidia* and *Telopsammis* is demonstrated by five synapomorphies identified by Gee and Huys (1991) and Huys (2009) including the presence of two rat tail-like apical setae on P1 enp-2, the absence of an inner seta on P3 enp-2 and P4 exp-3, the completely fused female P5 without a distinguishable division between the exopod and baseoendopodal lobe, and the presence of two setae on the male P6. The presence of only two setae on the male P6 is an advanced state compared with the presence of three setae in more primitive congeners bearing aesthetascs on the mouthparts (*Paradanielssenia*, *Peltisenia*, *Sentiropsis*, *Jonesiella* as well as *Micropsammis*). Additionally, the genera *Leptotachidia* and *Telopsammis* both possess the following features: (1) both members of female P5 are completely separated, with a small sclerite between them; and (2) the penultimate somite has no deeply incised frills. *Leptotachidia* can be considered the most advanced genus because of the following features: (1) presence of the Brodskaya organ on the terminal antennular segment; the Brodskaya organ is a densely opaque bulbous appendage that is a sensory organ for a bathyal existence; (2) female antennule five-segmented instead of six; (3) loss of sexual dimorphism in the male P2; and (4) the absence of an inner seta on P2–P4 exp-2 (Gee and Huys 1991).

Becker (1974) erected the genus *Leptotachidia* for *L. iberica* Becker, 1974, which was found at 3800 m depth off the Atlantic coast of Portugal. Upon careful re-examination of the type material, Gee and Huys (1991) identified some discrepancies between the original description and type specimens, and provided an amended diagnosis of the genus (Gee and Huys 1991: 1136). The two new species from the northwestern Pacific were placed into *Leptotachidia* based on Gee and Huys (1991) and on the autapomorphies of the genus mentioned above, except for rostrum and genital double-somite (see below). However, we were unable to confirm the male condition due to the lack of male specimens although the sexual dimorphic character could provide a significant evidence for the phylogenetic relationships among the danielsseniin genera (e.g. Gee and Huys 1990, 1991, 1994; Huys and Gee 1992, 1993, 1996a, b).

The most important character to discriminate both new Pacific species from the type species *L. iberica* is the setal armature of the thoracic legs (Table 1): (1) *L. senaria* sp. nov. possesses two elements on P2 enp-3 instead of three as in *L. iberica*, (2) P3–P4 enp-3 possesses a single apical element instead of three and two, respectively, as in *L. iberica*, (3) P3 exp-3 possesses two outer spines instead of three as in *L. iberica*, and (4) the female P5 possesses six elements instead of five as in *L. iberica*. In contrast, the reduced elements in P3 enp-3 of *L. apousia* sp. nov. possesses two setae only. In addition, the armature of the antennary exopod of *L. apousia* sp. nov. is unique by having a single seta on the proximal segment, whereas both *L. iberica* and *L. senaria* sp. nov. have two elements on the corresponding segment.

**Table 1.** Comparison of morphological characters among *Leptotachidia* species based on the female.

		*L. iberica*	*L. senaria* sp. nov.	*L. apousia* sp. nov.
No. of sensilla on Ro		1 pair	2 pairs	2 pairs
Sensilla form of distal margin on Ro		sensilla-like	ae-like	ae-like
No. of setae on A2 exp-1		2	2	1
Lateral seta on Md enp		delicate	rudimentary	absent
Claviform ae on Md, Mxl, and Mxa		naked	with spinules	with spinules
Seta on claw of Mxp enp		delicate	rudimentary	rudimentary
Setal armature	P2 enp-3	021	020	021
	P3 exp-3	123	122	123
	P3 enp-3	021	010	011
	P4 enp-3	011	010	011
	P5	5	6	5
Genital double-somite		fused completely	divided by ventral and lateral ridges	divided by ventral and lateral ridges (each separate)
CR L/W ratio		1.2	1.7	1.4
Reference		Becker 1974; Gee and Huys 1991	present study	present study

Abbreviation: A2, antenna; ae, aesthetasc; CR, caudal ramus; enp, endopod; L/W ratio, length to width ratio; Md, mandible; Mxa, maxilla; Mxl, maxillule; Mxp, maxilliped; Ro, rostrum

The two new species described here can be separated from *L. iberica* by (1) the presence of two pairs of sensilla on the rostrum, of which the distal ones modified into aesthetasc in the new species, but with one pair of normal sensilla on the rostrum of *L. iberica*, (2) peduncle of the claviform aesthetasc of the mouthparts with spinules in the new species, but naked in *L. iberica*, (3) accessory seta on the maxillipedal claw rudimentary in the new species, but clearly visible and well developed in *L. iberica*, and (4) both halves of genital double-somite separated by surface ridges laterally and ventrally, but completely fused in *L. iberica*. However, the two new species are clearly distinguishable from each other by the setal armature of the antennary exopod and thoracic legs, the structure and shape of the genital double-somite (ventral and lateral surface ridges are clearly separate in *L. apousia* sp. nov., but they are continued in *L. senaria* sp. nov.), the lateral armature of the mandibular endopod (with rudimentary seta in *L. senaria* sp. nov., but unarmed in *L. apousia* sp. nov.), and the length/width-ratio of the caudal ramus (about 1.7 times in *L. senaria* sp. nov. vs. 1.4 in *L. apousia* sp. nov.).

Members of the family Pseudotachidiidae are important component of harpacticoid assemblages in deep-sea habitats (Seifried 2004; Willen and Schulz 2007; Willen 2008, 2009; Kitahashi et al. 2012; George et al. 2014). Within the group of pseudotachidiids bearing aesthetascs on the mouthparts, only two species, *L. iberica* and *Nyxis rostrocularis* Willen, 2009, have been recorded from abyssal habitats (Becker 1974; Willen 2009). Many abyssal harpacticoids show extraordinary adaptations to deep-sea habitats (Seifried and Schminke 2003; Mercado-Salas et al. 2019), one of which is the Brodskaya organ which serves as a sensory structure on the antennule in both sexes, as in *L. iberica* and in the deep-sea *Cerviniopsis obtusirostris* Brotzkaja, 1963 (Aegisthidae) (Por 1969; Gee and Huys 1991). However, Gee and Huys (1991) suggested that the Brodskaya organ of *L. iberica* and *C. obtusirostris* is not homologous based on the morphological or phylogenetic discrepancies. Within *Cerviniopsis* Sars, 1903, only *C. obtusirostris* possesses this organ, suggesting that the presence of this structure in Pseudotachidiidae and Aegisthidae is probably a result of convergent evolution. The presence of the Brodskaya organ in the antennule is an important autapomorphy supporting the monophyly of *Leptotachidia* within the family, including two new *Leptotachidia* species from the Pacific deep sea. We also identified an additional sensory structure in both *L. senaria* sp. nov. and *L. apousia* sp. nov., that is, a pair of distal sensilla on the rostrum modified into aesthetascs (Figs 2C, 6C). Although Gee and Huys (1991) depicted the corresponding structures of *L. iberica* as nominal sensilla, we believe that this modification in both Pacific species is also a result of adaptation to the deep sea.

Despite the high abundance and species diversity of abyssal harpacticoids, few taxonomical studies are available (Seifried 2004). Our knowledge on the distribution of the genus *Leptotachidia* was limited to the deep sea of the Atlantic Ocean (Becker 1974; Gee and Huys 1991). The record of the two new species co-occurring in the same subsample presented herein expands the distribution range of the genus to the northwestern Pacific in abyssal sediments at 5482 m depth suggesting that *Leptotachidia* species display a wider geographical distribution than previously thought, and that the scant record of the genus is due to a lack of sampling and to the lack of taxonomical studies in abyssal habitats.

### Key to species of the genus *Leptotachidia* Becker, 1974

**Table:** 

1	P3 exp-3 with three outer spines; female P5 with five elements in all	**2**
–	P3 exp-3 with two outer spines; female P5 with six elements in all	***L. senaria* sp. nov.**
2	Proximal segment of the antennary exopod with two setae; P3 enp-3 with three elements	***L. iberica* Becker, 1974**
–	Proximal segment of the antennary exopod with one seta; P3 enp-3 with two elements	***L. apousia* sp. nov.**

## Supplementary Material

XML Treatment for Leptotachidia

XML Treatment for Leptotachidia senaria

XML Treatment for Leptotachidia apousia
